# Spatiotemporal distribution of a non-haematophagous bat community and rabies virus circulation: a proposal for urban rabies surveillance in Brazil

**DOI:** 10.1017/S0950268818003229

**Published:** 2019-03-08

**Authors:** R. A. Dias, F. Rocha, F. M. Ulloa-Stanojlovic, A. Nitsche, C. Castagna, T. de Lucca, R. C. A. Rodrigues

**Affiliations:** 1 Department of Preventive Veterinary Medicine and Animal Health, School of Veterinary Medicine, University of Sao Paulo, Sao Paulo, Brazil; 2 Unidade de Vigilância de Zoonoses de Campinas, Prefeitura Municipal de Campinas, Campinas, Brazil; 3 Vigilância em Saúde, Prefeitura Municipal de Campinas, Campinas, Brazil

**Keywords:** Control, non-haematophagous bat, rabies, relative risk, spatiotemporal

## Abstract

In Brazil, rabies surveillance is based on monitoring domestic and wild animals, although the most prevalent lineage of the rabies virus (RABV) currently diagnosed in Brazil is associated with bats, particularly non-haematophagous bats. Disease control is based on the mass vaccination of dogs and cats. We used data collected by the passive surveillance system of the city of Campinas from 2011 to 2015, to describe the temporal and geographic distributions of the bat specimens and RABV and discuss the current rabies surveillance with the advent of the declaration of canine and feline rabies-free areas in Brazil. We described the species, locations and health statuses of the collected bat specimens. Moreover, all samples were submitted for RABV diagnosis. Then, we performed a time series decomposition for each bat family. Additionally, we determined the spatiotemporal relative risk for RABV infection using the ratio of the kernel-smoothed estimates of spatiotemporal densities of RABV-positive and RABV-negative bats. From the 2537 bat specimens, the most numerous family was Molossidae (72%), followed by Vespertilionidae (14%) and Phyllostomidae (13%). The bat families behaved differently in terms of seasonal and spatial patterns. The distribution of bats varied geographically in the urban environment, with Molossidae and Phyllostomidae being observed downtown and Vespertilionidae being observed in peripheral zones. Concurrently, a significant relative risk of RABV infection was observed downtown for Vespertilionidae and in peripheral zones for Molossidae. No RABV-positive sample clusters were observed. As a result of the official declaration of RABV-free areas in southern Brazil, mass dog and cat vaccinations are expected to halt in the near future. This stoppage would make most dog and cat populations susceptible to other RABV lineages, such as those maintained by non-haematophagous bats. In this scenario, all information available on bats and RABV distribution in urban areas is essential. Currently, few studies have been conducted. Some local health authorities, such as that in Campinas, are spontaneously basing their surveillance efforts on bat rabies, which is the alternative in reality scenario of increased susceptibility to bat-associated RABV that is developing in Brazil.

## Introduction

Bats are among the taxa that can adapt to the urban environment [1–3]. Of the 178 bat species recorded in Brazil [4], at least 84 (47.2%) occur in urban areas [5]. Generalist species may benefited from the availability of artificial roosts and food items, such as fruits from urban trees and insects attracted by the city lights [5]. The increased abundance of bats in urban areas may also generate conflicts with humans and domestic animals, both as a nuisance and, more importantly, in the transmission of diseases, particularly those caused by RNA viruses, including the rabies virus (RABV), Ebola virus, SARS-CoV, Hendra virus and Nipah virus [6, 7]. According to the United Nations, currently 87% of the Brazilian population lives in urban areas, a proportion higher than that observed in the United States and United Kingdom [8], but few studies address the bat fauna in Brazilian cities, and even fewer address their pathogens [5, 9].

Rabies is a cosmopolitan zoonotic disease caused by a virus from the family Rhabdoviridae in the genus *Lyssavirus* transmitted among mammals via bites and the subsequent inoculation of the virus in the saliva of infected hosts [10]. Several lineages or antigenic variants (AgV) of the RABV occur that are adapted to specific taxa, such as carnivores, bats or non-human primates [11]. Spillover of these specific lineages, including bat-associated lineages infecting other mammals, to other taxa is common [6, 12].

Although the most prevalent lineages of RABV currently diagnosed in Brazil are associated with bats, specifically non-haematophagous bats [11, 13], the national-level human rabies surveillance and control remain based on the transmission of AgV1 and AgV2, both of which are associated with carnivores, mostly dogs and cats. The rabies surveillance system performed by local health authorities is based on monitoring the occurrence of rabies in domestic animals by surveilling deceased and euthanised dogs showing neurological symptoms in public shelters, until the sample reaches 0.2% of the estimated dog population [14, 15]. Rabies control is based on the mass vaccination of at least 80% of the estimated dog and cat populations, clinical observation of dogs and cats that bite a human for at least 10 days after the attack, post-exposure vaccination for the attack victims and emergency vaccination of all unvaccinated dogs and cats within a 5 km buffer area around a suspicious case [14, 15]. This effort resulted in a declining trend of human rabies, with few cases remaining in north and northeast Brazil yearly.

Campinas, located 96 km northwest of Sao Paulo City, is one of the largest cities in Brazil, with ~1 million inhabitants [16]. The city can be considered as a case study for rabies surveillance and control for the rest of Brazil. Campinas has an Animal Control Authority (UVZ) that has been in place since 1972, when mass dog and cat vaccination campaigns started. In 2009, 90 085 dogs and 9001 cats were vaccinated against RABV [15]. In 2010, the mass vaccination campaign was halted because of vaccine accidents, followed by a reduction in the delivery of rabies vaccines for dogs and cats from the Brazilian Ministry of Health. At that moment, only emergency vaccinations of dogs and cats inside a buffer area of 5 km (i.e. blockage area) around suspicious or confirmed dog or cat rabies cases could be performed. The mass vaccination campaigns resumed in 2012, were interrupted in 2015, and resumed again in 2016. However, after these changes, two cats and a dog with rabies demonstrated to be bat variant rabies [17].

Although the UVZ historically focused on rabies control by the mass vaccination of dogs and cats against RABV, a passive surveillance system for bats was implemented in 1994. Reports of bat presence are received through a call centre, and the UVZ staff goes immediately to the location and collects dead or alive bat specimens and sends them for RABV diagnosis. The number of notifications increased slowly until 2010, when 488 bats were captured [15]. Since then, a mean number of 536 bats were retrieved from Campinas households yearly (personal communication).

Unlike the other Brazilian cities, in Campinas when a bat from the passive surveillance system is diagnosed with RABV, house-to-house communication and distribution of educational material (including the call centre number for further notifications) begin immediately in the area in which the positive bat was found. During these visits, the number of unvaccinated dogs and cats are recorded and their owners are encouraged to vaccinate them.

Currently, the probability of bat-associated RABV to be transmitted from bats to dogs and cats and then to humans is higher than that of carnivore-associated RABV (AgV1 and AgV2) to be transmitted from dogs and cats to humans in Brazil. Therefore, if the vaccine protection declines with the declaration of AgV1- and AgV2-free areas in Southern Brazil in 2015, dogs and cats will become susceptible to bat-associated RABV. Few studies described bat communities in urban areas [18, 19], with some studies describing the effects of city lights [20] and lead poisoning [21] over bats in urban areas. In South America, a single study aimed at the description of bat communities in urban areas of Chile [22], and two Brazilian studies aimed at the investigation of *Candida* sp. [23] and *Leptospira* spp. [24]. The aim of this study was to describe the bat community and RABV distributions from data collected by the Campinas passive surveillance system from 2011 to 2015 to elucidate key points regarding the rabies surveillance in a future scenario of dogs and cats susceptibility to bat-associated RABV.

## Materials and methods

### Bat passive surveillance database

The study region was the city of Campinas, Sao Paulo State, Brazil. The city has a bat rabies passive surveillance system in place which collects all dead and alive bats found in the households. Data from the UVZ bat rabies passive surveillance were compiled from 2011 to 2015 and included the species, address, geographic coordinates, date, location of the bat (inside, outside the building or household) and health status (dead or alive) of the bats. The UVZ staff identified the bat species, and the identification was confirmed by the Pasteur Institute of Sao Paulo, to which the specimens were sent for RABV diagnosis. Bats were submitted to a direct immunofluorescence test and RABV antigenic analysis. The RABV qualitative results (positive and negative) were kindly made available for this work. The proportions of RABV-positive samples and associated 95% confidence intervals (CI) in each bat family were calculated using prop.test( ) function of R.

The UVZ only began only to compile information about bats that directly or indirectly contacted dogs and cats in 2015. However, this type of record was not available when bats were found in the street or public areas. Since 2011, three rabies cases were recorded in Campinas: a cat infected with the *Nyctinomops* sp. RABV lineage in 2014; a dog infected with the *Desmodus rotundus* lineage (AgV3) in 2015; and a cat infected with the *Myotis* sp. lineage in 2016 [17]. All reported data had their geographic coordinates associated, which allowed locating them on thematic maps and perform spatial analyses.

### General description of the collected bat specimens

For each bat family, genus and species, the general characteristics (sex, reproductive status) and the circumstances in which the specimens were found (capture place and health status) were described in proportions. The reproductive status (mature or immature) was defined by the presence of testes in males and mammary glands and nipples in the subaxillary region in females [25]. In order to describe the bat traits, first we compared the proportions of each variable between the bat families and then across the genera within each family using the prop.test( ) function of R.

The list of bat species collected during the study period by the Campinas UVZ was compared with the Integrated Taxonomic Information System database (itis.gov) to verify their worldwide occurrence. The areas of occurrence of all species were verified in the SpeciesLink Network database (splink.org.br), which is a database of Brazilian animals and plants deposited in 456 collections worldwide, to verify that the collected bat specimens were likely to be found in Campinas.

The biological features (size, weight, colony size, feeding habits, reproduction and roost type) of the bat species collected during the study period and those not collected but likely to be found in Campinas were compiled from the available literature [5, 26–29].

### Temporal distribution of collected bat specimens

The time series for each bat family time series was decomposed to determine the existence of seasonal or trend patterns during the study period. The seasonality period was detected via periodogram analysis, using Fournier transformation in the *TSA* package of R. We have chosen the additive model because the magnitude of the seasonal component was stable in the time series and used the decompose( ) function.

### Spatiotemporal analysis of collected bat specimens and RABV

Regardless of the RABV laboratory results, the geographic distribution of *Cynomops planirostris*, *Eumops glaucinus*, *Molossus molossus*, *Tadarida brasiliensis*, *Artibeus lituratus*, *Glossophaga soricina*, *Eptesicus* spp. and *Myotis* spp., each with more than 100 collected specimens during the study period were represented in thematic maps, using QGIS software. *Eptesicus* spp. and *Myotis* spp. were grouped due to their similar traits for this geographic representation. Additionally, using the distribution of bat specimens, a kernel-smoothing with a 10 km bandwidth radius was performed using the Heat Map plugin of QGIS, and contour lines were extracted using the Extraction plugin of QGIS. This bandwidth was chosen due to the maximum foraging distance of frugivorous and haematophagous bats [26].

In order to verify the existence of clustering of RABV-positive samples, the generalisation of the Ripley's K function for bivariate spatial point processes, or the difference between the cumulative distribution functions of distances from random points to nearest RABV-positive and RABV-negative samples was calculated using the *splancs* package of R. Moreover, an envelope of the 2.5% and 97.5% quantiles of the difference function was estimated from 999 Monte Carlo simulations. RABV-positive sample clusters were identified when the difference function crossed the positive values of its associated envelope [30]. The analysis was made for the species and genera with more than 100 collected specimens (described above).

The Campinas urban zoning map [31] was used to stratify the locations of the collected specimens into five land uses: residential, commercial, industrial, protected area (forest fragments) and rural area. The residential strata were subdivided into residential (unifamiliar households) and apartments (multifamiliar buildings). The proportions of bat species or genera with more than 100 collected specimens (described above) in each urban zoning stratum were compared using the prop.test( ) function of R.

The spatiotemporal relative risk for RABV infection was obtained by the ratio of the kernel-smoothed estimates of spatiotemporal densities of RABV-positive and RABV-negative bats in February (mid-summer), May (mid-autumn), August (mid-winter) and November (mid-spring) using the *sparr* package of R [32]. Data from the different years were stratified by months and the density ratio was calculated using the spattemp.density ( ) function considering a fixed bandwidth kernel estimate of the continuous spatiotemporal data. The asymptotic tolerance contours (*P* = 0.05 and *P* = 0.001) were obtained to identify significantly increased relative risk areas.

## Results

### General description of the collected bat specimens

During the study period, the Campinas UVZ collected 2537 bat specimens of 25 different species in 16 genera (*Cynomops*, *Eumops*, *Molossops*, *Molossus*, *Nyctinomops*, *Promops*, *Tadarida*, *Artibeus*, *Glossophaga*, *Phyllostomus*, *Platyrrhinus*, *Sturnira*, *Eptesicus*, *Histiotus*, *Lasiurus* and *Myotis*) and three families (Molossidae, Phyllostomidae and Vespertilionidae) (Table 1). With 1835 collected specimens (72%), Molossidae was the most numerous family, followed by Vespertilionidae (*n* = 348 or 14%) and Phyllostomidae (*n* = 325 or 13%). A few specimens (*n* = 29 or 1%) were not identified. The most numerous species (*n* > 100) were *M. molossus* (*n* = 873), *C. planirostri*s (*n* = 175), *E. glaucinus* (*n* = 146), *T. brasiliensis* (*n* = 140) and *G. soricina* (*n* = 109).

**Table 1.** General characteristics and circumstances that collected bats have been found from 2011 to 2015 in Campinas, Brazil

Family genus and species*	Sex			Reproductive status			Capture place			Health status			Total
	Female (%)	Male (%)	? (%)	Immature (%)	Mature (%)	? (%)	Inside (%)	Outside (%)	? (%)	Dead (%)	Alive (%)	? (%)	
**Molossidae**	**783** (**43)**	**979** (**53)**	**73** (**4)**	**332** (**18)**	**1311** (**71)**	**192** (**11)**	**844** (**46)**	**596** (**32)**	**395** (**22)**	**656** (**36)**	**1059** (**58)**	**120** (**6)**	**1835**
**Cynomops**	**84** (**45)**	**97** (**52)**	**5** (**3)**	**53** (**28)**	**113** (**61)**	**20** (**11)**	**78** (**42)**	**61** (**33)**	**47** (**25)**	**80** (**43)**	**89** (**48)**	**17** (**9)**	**186**
* C. abrasus*	3 (50)	3 (50)	0	2 (33)	4 (67)	0	4 (66)	1 (17)	1 (17)	3 (50)	2 (33)	1 (17)	**6**
* C. planirostris*	78 (45)	93 (53)	4 (2)	49 (28)	108 (62)	18 (10)	72 (41)	57 (33)	46 (26)	72 (41)	87 (50)	16 (9)	**175**
* Cynomops* spp. (2^a^/5)	3 (60)	1 (20)	1 (20)	2 (40)	1 (20)	2 (40)	2 (40)	3 (60)	0	5 (100)	0	0	**5**
**Eumops**	**100** (**48)**	**100** (**48)**	**10** (**4)**	**31** (**15)**	**161** (**77)**	**18** (**8)**	**87** (**41)**	**81** (**39)**	**42** (**20)**	**66** (**32)**	**129** (**61)**	**15** (**7)**	**210**
* E. glaucinus*	73 (50)	69 (47)	4 (3)	24 (17)	113 (77)	9 (6)	48 (33)	65 (44)	33 (23)	47 (32)	88 (60)	11 (8)	**146**
* E. perotis*	22 (41)	28 (53)	3 (6)	5 (9)	40 (76)	8 (15)	37 (70)	10 (19)	6 (11)	10 (19)	39 (73)	4 (8)	**53**
* Eumops* spp. (5^b^/16)	5 (46)	3 (27)	3 (27)	2 (18)	8 (73)	1 (9)	2 (18)	6 (55)	3 (27)	9 (82)	2 (18)	0	**11**
**Molossops**	**0**	**2** (**67)**	**1** (**33)**	**1** (**33)**	**1** (**33)**	**1** (**34)**	**2** (**67)**	**1** (**33)**	**0**	**0**	**3** (**100)**	**0**	**3**
* Molossops* sp. (1^c^/4)	0	2 (67)	1 (33)	1 (33)	1 (33)	1 (34)	2 (67)	1 (33)	0	0	3 (100)	0	**3**
**Molossus**	**396** (**40)**	**566** (**57)**	**24** (**3)**	**198** (**20)**	**701** (**71)**	**87** (**9)**	**420** (**43)**	**348** (**35)**	**218** (**22)**	**355** (**36)**	**554** (**56)**	**77** (**8)**	**986**
* M. molossus*	357 (41)	493 (56)	23 (3)	180 (21)	618 (71)	75 (8)	375 (43)	298 (34)	200 (23)	315 (36)	491 (56)	67 (8)	**873**
* M. rufus*	30 (34)	59 (66)	0	12 (13)	66 (74)	11 (13)	35 (39)	41 (46)	13 (15)	32 (36)	47 (53)	10 (11)	**89**
* Molossus* spp. (2^d^/9)	9 (38)	14 (58)	1 (4)	6 (25)	17 (71)	1 (4)	10 (42)	9 (38)	5 (20)	8 (33)	16 (67)	0	**24**
**Nyctinomops**	**25** (**47)**	**27** (**51)**	**1** (**2)**	**5** (**9)**	**42** (**80)**	**6** (**11)**	**35** (**66)**	**8** (**15)**	**10** (**19)**	**13** (**24)**	**40** (**76)**	**0**	**53**
* N. laticaudatus*	9 (50)	8 (44)	1 (6)	4 (22)	11 (61)	3 (17)	15 (83)	0	3 (17)	3 (17)	15 (83)	0	**18**
* Nyctinomops* spp. (3^e^/4)	16 (46)	19 (54)	0	1 (3)	31 (89)	3 (8)	20 (57)	8 (23)	7 (20)	10 (29)	25 (71)	0	**35**
**Promops**	**4** (**80)**	**1** (**20)**	**0**	**2** (**40)**	**3** (**60)**	**0**	**3** (**60)**	**1** (**20)**	**1** (**20)**	**3** (**60)**	**2** (**40)**	**0**	**5**
* Promops* sp. (1^f^/2)	4 (80)	1 (20)	0	2 (40)	3 (60)	0	3 (60)	1 (20)	1 (20)	3 (60)	2 (40)	0	**5**
**Tadarida**	**77** (**55)**	**61** (**44)**	**2** (**1)**	**16** (**11)**	**112** (**80)**	**12** (**9)**	**106** (**76)**	**11** (**8)**	**23** (**16)**	**24** (**17)**	**111** (**79)**	**5** (**4)**	**140**
* T. brasiliensis*	39 (59)	26 (39)	1 (2)	13 (20)	48 (73)	5 (7)	46 (70)	7 (11)	13 (19)	10 (15)	54 (82)	2 (3)	**66**
* Tadarida* sp. (1^g^/10)	38 (51)	35 (47)	1 (2)	3 (4)	64 (86)	7 (10)	60 (81)	4 (5)	10 (14)	14 (19)	57 (77)	3 (4)	**74**
**Not classified**	**97** (**38)**	**125** (**50)**	**30** (**12)**	**26** (**10)**	**178** (**71)**	**48** (**19)**	**113** (**45)**	**85** (**34)**	**54** (**21)**	**115** (**46)**	**131** (**52)**	**6** (**2)**	**252**
**Phyllostomidae**	**137** (**42)**	**166** (**51)**	**22** (**7)**	**27** (**8)**	**241** (**74)**	**57** (**18)**	**106** (**33)**	**141** (**43)**	**78** (**24)**	**206** (**63)**	**106** (**33)**	**13** (**4)**	**325**
**Artibeus**	**58** (**42)**	**73** (**53)**	**6** (**5)**	**16** (**12)**	**97** (**71)**	**24** (**17)**	**24** (**17)**	**91** (**67)**	**22** (**16)**	**89** (**65)**	**45** (**33)**	**3** (**2)**	**137**
* Artibeu lituratus*	31 (43)	38 (53)	3 (4)	10 (14)	50 (69)	12 (17)	13 (18)	49 (68)	10 (14)	43 (60)	26 (36)	3 (4)	**72**
* Artibeus* spp. (4^h^/12)	27 (41)	35 (54)	3 (5)	6 (9)	47 (73)	12 (18)	11 (17)	42 (65)	12 (18)	46 (71)	19 (29)	0	**65**
**Glossophaga**	**49** (**45)**	**54** (**49)**	**6** (**6)**	**6** (**6)**	**83** (**76)**	**20** (**18)**	**53** (**49)**	**22** (**20)**	**34** (**31)**	**67** (**61)**	**35** (**32)**	**7** (**7)**	**109**
* G. soricina*	30 (44)	38 (55)	1 (1)	5 (7)	51 (74)	13 (19)	34 (49)	15 (22)	20 (29)	42 (61)	23 (33)	4 (6)	**69**
* Glossophaga* sp. (1^i^/5)	19 (47)	16 (40)	5 (13)	1 (2)	32 (80)	7 (18)	19 (47)	7 (18)	14 (35)	25 (62)	12 (30)	3 (8)	**40**
**Phyllostomus**	**6** (**67)**	**3** (**33)**	**0**	**0**	**8** (**89)**	**1** (**11)**	**2** (**22)**	**5** (**56)**	**2** (**22)**	**4** (**44)**	**5** (**56)**	**0**	**9**
* Phyllostomus discolor*	3 (75)	1 (25)	0	0	3 (75)	1 (25)	1 (25)	2 (50)	1 (25)	3 (75)	1 (25)	0	**4**
* Phyllostomus* spp. (2^j^/4)	3 (60)	2 (40)	0	0	5 (100)	0	1 (20)	3 (60)	1 (20)	1 (20)	4 (80)	0	**5**
**Platyrrhinus**	**11** (**44)**	**13** (**52)**	**1** (**4)**	**1** (**4)**	**22** (**88)**	**2** (**8)**	**13** (**52)**	**5** (**20)**	**7** (**28)**	**14** (**56)**	**10** (**40)**	**1** (**4)**	**25**
* Platyrrhinus lineatus*	0	1 (100)	0	0	1 (100)	0	0	0	1 (100)	0	1 (100)	0	**1**
* Platyrrhinus* spp. (2^k^/11)	11 (46)	12 (50)	1 (4)	1 (4)	21 (88)	2 (8)	13 (54)	5 (21)	6 (25)	14 (58)	9 (38)	1 (4)	**24**
**Sturnira**	**2** (**40)**	**2** (**40)**	**1** (**20)**	**0**	**4** (**80)**	**1** (**20)**	**0**	**3** (**60)**	**2** (**40)**	**3** (**60)**	**2** (**40)**	**0**	**5**
* Sturnira lilium*	1 (33)	2 (67)	0	0	3 (100)	0	0	1 (33)	2 (67)	2 (67)	1 (33)	0	**3**
* Sturnira* spp. (2^l^/23)	1 (50)	0	1 (50)	0	1 (50)	1 (50)	0	2 (100)	0	1 (50)	1 (50)	0	**2**
**Not classified**	**11** (**27)**	**21** (**53)**	**8** (**20)**	**4** (**10)**	**27** (**67)**	**9** (**23)**	**14** (**35)**	**15** (**37)**	**11** (**28)**	**29** (**72)**	**9** (**23)**	**2** (**5)**	**40**
**Vespertilionidae**	**147** (**42)**	**171** (**49)**	**30** (**9)**	**101** (**29)**	**207** (**59)**	**40** (**12)**	**133** (**38)**	**143** (**41)**	**72** (**21)**	**169** (**49)**	**143** (**41)**	**36** (**10)**	**348**
**Eptesicus**	**48** (**47)**	**45** (**45)**	**8** (**8)**	**33** (**33)**	**62** (**61)**	**6** (**6)**	**37** (**37)**	**44** (**43)**	**20** (**20)**	**41** (**41)**	**47** (**46)**	**13** (**13)**	**101**
* Eptesicus brasiliensis*	2 (67)	1 (33)	0	2 (67)	1 (33)	0	0	2 (67)	1 (33)	2 (67)	1 (33)	0	**3**
* E. diminutus*	2 (67)	1 (33)	0	1 (33)	2 (67)	0	2 (67)	0	1 (33)	2 (67)	0	1 (33)	**3**
* E. furinalis*	13 (54)	10 (42)	1 (4)	10 (42)	13 (54)	1 (4)	6 (25)	10 (42)	8 (33)	10 (42)	8 (33)	6 (25)	**24**
* E. fuscus*	2 (25)	4 (50)	2 (25)	3 (37)	3 (38)	2 (25)	2 (25)	4 (50)	2 (25)	4 (50)	0	4 (50)	**8**
* Eptesicus* spp. (4^m^/24)	29 (46)	29 (46)	5 (8)	17 (27)	43 (68)	3 (5)	27 (43)	28 (44)	8 (13)	23 (37)	38 (60)	2 (3)	**63**
**Histiotus**	**8** (**50)**	**7** (**44)**	**1** (**6)**	**4** (**25)**	**10** (**63)**	**2** (**12)**	**7** (**44)**	**7** (**44)**	**2** (**12)**	**7** (**44)**	**9** (**56)**	**0**	**16**
* Histiotus velatus*	5 (56)	3 (33)	1 (11)	4 (44)	4 (45)	1 (11)	3 (33)	5 (56)	1 (11)	4 (44)	5 (56)	0	**9**
* Histiotus* sp. (1^n^/7)	3 (43)	4 (57)	0	0	6 (86)	1 (14)	4 (57)	2 (29)	1 (14)	3 (43)	4 (57)	0	**7**
**Lasiurus**	**6** (**38)**	**9** (**56)**	**1** (**6)**	**2** (**12)**	**13** (**82)**	**1** (**6)**	**6** (**37)**	**6** (**38)**	**4** (**25)**	**6** (**37)**	**6** (**38)**	**4** (**25)**	**16**
* Lasiurus blosevilli*	1 (25)	3 (75)	0	1 (25)	3 (75)	0	3 (75)	1 (25)	0	3 (75)	0	1 (25)	**4**
* L. cinereus*	1 (100)	0	0	0	1 (100)	0	0	1 (100)	0	0	1 (100)	0	**1**
* L. ega*	1 (100)	0	0	1 (100)	0	0	0	1 (100)	0	0	1 (100)	0	**1**
* Lasiurus* spp. (3^o^/17)	3 (30)	6 (60)	1 (10)	0	9 (90)	1 (10)	3 (30)	3 (30)	4 (40)	3 (30)	4 (40)	3 (30)	**10**
**Myotis**	**73** (**42)**	**92** (**53)**	**9** (**5)**	**52** (**30)**	**103** (**59)**	**19** (**11)**	**70** (**40)**	**66** (**38)**	**38** (**22)**	**85** (**49)**	**70** (**40)**	**19** (**11)**	**174**
* M. albescens*	3 (60)	2 (40)	0	2 (40)	2 (40)	1 (20)	4 (80)	1 (20)	0	1 (20)	4 (80)	0	**5**
* M. nigricans*	37 (49)	36 (47)	3 (4)	38 (50)	33 (43)	5 (7)	33 (43)	22 (29)	21 (28)	34 (45)	29 (38)	13 (17)	**76**
* Myotis* spp. (5^p^/118)	33 (36)	54 (58)	6 (6)	12 (13)	68 (73)	13 (14)	33 (36)	43 (46)	17 (18)	50 (54)	37 (40)	6 (6)	**93**
**Not classified**	**12** (**29)**	**18** (**44)**	**11** (**27)**	**10** (**25)**	**19** (**46)**	**12** (**29)**	**13** (**32)**	**20** (**49)**	**8** (**19)**	**30** (**73)**	**11** (**27)**	**0**	**41**
**Not classified**	**1** (**3)**	**2** (**7)**	**26** (**90)**	**0**	**2** (**7)**	**27** (**93)**	**13** (**44)**	**8** (**28)**	**8** (**28)**	**14** (**48)**	**7** (**24)**	**8** (**28)**	**29**
**Total**	**1068** (**42)**	**1318** (**52)**	**151** (**6)**	**460** (**18)**	**1761** (**69)**	**316** (**13)**	**1096** (**43)**	**888** (**35)**	**553** (**22)**	**1045** (**41)**	**1315** (**52)**	**177** (**7)**	**2537**

*Numbers between parentheses indicate the number of possible bat species in Campinas out of the total number of worldwide extant bat species of each genus.

a *Cynomops abrasus* and *C. planirostris*.

b *Eumops auripendulus*, *E. bonariensis*, *E. glaucinus*, *E. hansae* and *E. perotis*.

c All specimens of this genus were likely to be *Molossops temminckii*.

d *M. molossus* and *M. rufus*.

e *Nyctinomops aurispinosus*, *N. laticaudatus* and *N. macrotis*.

f All specimens of this genus were likely to be *Promops nasutus*.

g All specimens of this genus are likely to be *T. brasiliensis*.

h *Artibeus fimbriatus*, *A. lituratus*, *A. obscurus* and *A. planirostris*.

i All specimens of this genus were likely to be *G. soricina*.

j *P. discolor* and *P. hastatus*.

k *P. lineatus* and *P. recifinus*.

l *S. lilium* and *S. tildae*.

m *E. brasiliensis*, *E. diminutus*, *E. furinalis* and *E. fuscus*.

n All specimens of this genus were likely to be *H. velatus*.

o *L. blosevillii*, *L. cinereus* and *L. ega*.

p *M. albescens*, *M. levis*, *M. nigricans*, *M. riparius* and *M. simus*.

Most of the specimens were male (52%), and similar proportions of genera were observed between families, although among Molossidae, a higher proportion of females were observed in *Eumops* (48%; *P* = 0.02) and *Tadarida* (55%; *P* = 0.001) than in *Molossus* (40%). For the reproductive status, most of the specimens were mature (69%), but the proportions of immature of Molossidae (18%; *P* < 0.001) and Vespertilionidae (29%; *P* < 0.001) was significantly higher than that of Phyllostomidae (8%). With the Phyllostomidae and Vespertilionidae families, similar proportions of reproductive statuses were observed among genera, but within Molossidae, the proportion of immature *Cynomops* (28%) was higher than that of *Eumops* (15%; *P* < 0.001), *Molossus* (20%; *P* = 0.005), *Nyctinomops* (9%; *P* = 0.004) and *Tadarida* (11%; *P* < 0.001), and the proportion of immature *Molossus* was higher than that of *Tadarida* (*P* = 0.01).

Forty-three percent of the total specimens were captured inside households, but Phyllostomidae (33%; *P* < 0.001) and Vespertilionidae (38%; *P* < 0.001) were more frequently captured outside the households than Molossidae (46%). Within Molossidae, *Nyctinomops* (66%) were more frequently captured inside households than *Cynomops* (42%; *P* = 0.003), *Eumops* (41%; *P* < 0.001) and *Molossus* (43%; *P* < 0.001), and *Tadarida* (76%) were more frequently captured inside households than *Cynomops* (*P* < 0.001), *Eumops* (*P* < 0.001) and *Molossus* (*P* < 0.001). Within Phyllostomidae, *Glossophaga* (49%; *P* < 0.001) and *Platyrrhinus* (52%; *P* < 0.001) were more frequently captured inside households than *Artibeus* (17%). Similar proportions of reproductive statuses were observed among the different genera of Vespertilionidae.

Concerning the health status, most of the specimens were captured alive (52%). Nevertheless, Phyllostomidae (63%) were more frequently captured dead than Molossidae (36%; *P* < 0.001) and Vespertilionidae (49%; *P* < 0.001), and Vespertilionidae were more frequently captured dead than Molossidae (*P* < 0.001). Within Phyllostomidae and Vespertilionidae, similar proportions of health statuses were observed among genera, but within Molossidae, *Cynomops* (43%) were more frequently captured dead than *Eumops* (32%; *P* = 0.01), *Molossus* (36%; *P* = 0.04), *Nyctinomops* (24%; *P* = 0.003) and *Tadarida* (17%; *P* < 0.001). *Eumops* were more frequently captured dead than *Tadarida* (*P* = 0.001), and *Molossus* were more frequently captured dead than *Nyctinomops* (*P* = 0.03) and *Tadarida* (*P* < 0.001).

For the RABV diagnosis (Table 2), only 45 specimens tested positive (1.8%; 95% CI 1.3%–2.4%; Table 2). The proportions of positive samples of Phyllostomidae (6.5%; 95% CI 4%–9.7%) and Vespertilionidae (4%; 95% CI 2.2%–6.7%) were similar but higher than that in Molossidae (0.5%; 95% CI 0.3%–1%). RABV-positive samples were found in only six genera, with the highest proportion observed in *Artibeus* (15.3%), followed by *Myotis* (5.7%), *Eptesicus* (4%), *Tadarida* (3.6%), *Nyctinomops* (1.9%) and *Molossus* (0.4%). A total of 90 samples (3.5%) could not be submitted for RABV diagnosis and were designated bad samples, but when possible, features such as bat species, general characteristics and circumstances in which the specimens have been found were used in the analysis.

**Table 2.** RABV diagnosis in bats collected from 2011 to 2015 by a rabies passive surveillance system in Campinas, Brazil

Family genus and species	RABV diagnosis				Total
	Negative (%)	Positive (%)	95% CI*	No result (%)	
**Molossidae**	**1774** (**96.7)**	**10 (0.5)**	**0**.**3–1**.**0**	**51 (2.8)**	**1835**
**Cynomops**	**182** (**97.8)**	**0**	**0**.**0–2**.**0**	**4 (2.2)**	**186**
* C. abrasus*	6 (100)	0	0.0–46.0	0	**6**
* C. planirostris*	171 (97.7)	0	0.0–2.0	4 (2.3)	**175**
* Cynomops* spp.	5 (100)	0	0.0–52.2	0	**5**
**Eumops**	**204** (**97.1)**	**0**	**0**.**0–1**.**7**	**6 (2.9)**	**210**
* E. glaucinus*	142 (97.3)	0	0.0–2.5	4 (2.7)	**146**
* E. perotis*	52 (98.1)	0	0.0–6.7	1 (1.9)	**53**
* Eumops* spp.	10 (90.9)	0	0.0–28.5	1 (9.1)	**11**
**Molossops**	**3** (**100)**	**0**	**0**.**0–70**.**7**	**0**	**3**
* Molossops* sp.	3 (100)	0	0.0–70.7	0	**3**
**Molossus**	**964** (**97.8)**	**4 (0.4)**	**0**.**1–1**.**0**	**18 (1.8)**	**986**
* M. molossus*	855 (97.9)	4 (0.5)	0.1–1.2	14 (1.6)	**873**
* M. rufus*	86 (96.6)	0	0.0–4.0	3 (3.4)	**89**
* Molossus* spp.	23 (95.8)	0	0.0–14.0	1 (4.2)	**24**
**Nyctinomops**	**52** (**98.1)**	**1 (1.9)**	**0**.**0–10**.**0**	**0**	**53**
* N. laticaudatus*	17 (94.4)	1 (5.6)	0.1–27.3	0	**18**
* Nyctinomops* spp.	35 (100)	0	0.0–10.0	0	**35**
**Promops**	**5** (**100)**	**0**	**0**.**0–52**.**2**	**0**	**5**
* Promops* sp.	5 (100)	0	0.0–52.2	0	**5**
**Tadarida**	**135** (**96.4)**	**5 (3.6)**	**1**.**2–8**.**1**	**0**	**140**
* T. brasiliensis*	64 (97.0)	2 (3.0)	0.4–10.5	0	**66**
* Tadarida* sp.	71 (95.9)	3 (4.1)	0.8–11.4	0	**74**
**Not classified**	**229** (**90.9)**	**0**	**0**.**0–1**.**4**	**23 (9.1)**	**252**
**Phyllostomidae**	**284** (**87.4)**	**21 (6.5)**	**4**.**0–9**.**7**	**20 (6.1)**	**325**
**Artibeus**	**108** (**78.8)**	**21 (15.3)**	**9**.**7–22**.**5**	**8 (5.9)**	**137**
* A. lituratus*	58 (80.5)	12 (16.7)	8.9–27.3	2 (2.8)	**72**
* Artibeus* spp.	50 (76.9)	9 (13.9)	6.5–24.7	6 (9.2)	**65**
**Glossophaga**	**102** (**93.6)**	**0**	**0**.**0–3**.**3**	**7 (6.4)**	**109**
* G. soricina*	66 (95.6)	0	0.0–5.2	3 (4.4)	**69**
* Glossophaga* sp.	36 (90.0)	0	0.0–8.8	4 (10.0)	**40**
**Phyllostomus**	**9** (**100)**	**0**	**0**.**0–33**.**6**	**0**	**9**
* P. discolor*	4 (100)	0	0.0–60.2	0	**4**
* Phyllostomus* spp.	5 (100)	0	0.0–52.2	0	**5**
**Platyrrhinus**	**25** (**100)**	**0**	**0**.**0–13**.**7**	**0**	**25**
* P. lineatus*	1 (100)	0	0.0–97.5	0	**1**
* Platyrrhinus* spp.	24 (100)	0	0.0–14.2	0	**24**
**Sturnira**	**5** (**100)**	**0**	**0**.**0–52**.**2**	**0**	**5**
* S. lilium*	3 (100)	0	0.0–70.7	0	**3**
* Sturnira* spp.	2 (100)	0	0.0–84.2	0	**2**
**Not classified**	**35** (**87.5)**	**0**	**0**.**0–8**.**8**	**5 (12.5)**	**40**
**Vespertilionidae**	**322** (**92.5)**	**14 (4.0)**	**2**.**2–6**.**7**	**12 (3.5)**	**348**
**Eptesicus**	**94** (**93.0)**	**4 (4.0)**	**1**.**1–9**.**8**	**3 (3.0)**	**101**
* E. brasiliensis*	2 (66.7)	1 (33.3)	0.8–90.6	0	**3**
* E. diminutus*	2 (66.7)	1 (33.3)	0.8–90.6	0	**3**
* E. furinalis*	24 (100)	0	0.0–14.2	0	**24**
* E. fuscus*	8 (100)	0	0.0–36.9	0	**8**
* Eptesicus* spp.	58 (92.1)	2 (3.2)	0.4–11.0	3 (4.7)	**63**
**Histiotus**	**16** (**100)**	**0**	**0**.**0–20**.**6**	**0**	**16**
* H. velatus*	9 (100)	0	0.0–33.6	0	**9**
* Histiotus* sp.	7 (100)	0	0.0–41.0	0	**7**
**Lasiurus**	**16** (**100)**	**0**	**0**.**0–20**.**6**	**0**	**16**
* L. blosevilli*	4 (100)	0	0.0–60.7	0	**4**
* L. cinereus*	1 (100)	0	0.0**–**97.5	0	**1**
* L. ega*	1 (100)	0	0.0**–**97.5	0	**1**
* Lasiurus* spp.	10 (100)	0	0.0**–**30.8	0	**10**
**Myotis**	**158** (**90.8)**	**10 (5.7)**	**2**.**8–10**.**3**	**6 (3.5)**	**174**
* M. albescens*	4 (80.0)	1 (20.0)	0.5**–**71.6	0	**5**
* M. nigricans*	70 (92.1)	4 (5.3)	1.4**–**12.9	2 (2.6)	**76**
* Myotis* spp.	84 (90.3)	5 (5.4)	1.8**–**12.1	4 (4.3)	**93**
**Not classified**	**38** (**92.7)**	**0**	**0**.**0–8**.**6**	**3 (7.3)**	**41**
**Not classified**	**22** (**75.9)**	**0**	**0**.**0–11**.**9**	**7 (24.1)**	**29**
**Total**	**2402** (**94.7)**	**45 (1.8)**	**1**.**3–2**.**4**	**90 (3.5)**	**2537**

*Confidence interval, calculated through the binomial exact approach.

Table 3 presents a compilation of biological features of the bat species collected and those likely to occur in Campinas. Phyllostomidae have a longer forearm length and are heavier than Molossidae and Vespertilionidae. Molossidae and Vespertilionidae have a strict diet (general insectivores and aerial insectivores, respectively), whereas Phyllostomidae have a generalist diet (from insectivore, to frugivore, nectivore, pollinator, carnivore and flower eaters). Phyllostomidae have a more homogenous reproductive pattern (mostly poliestric) and fewer progeny (in general, one pup), whereas Molossidae and Vespertilionidae have diverse reproductive patterns (monoestric and poliestric), with Vespertilionidae producing more progeny (greater than two pups, in general). Molossidae and Vespertilionidae have higher proportions of species that can use artificial roosts (87% and 83%, respectively) than Phyllostomidae (58%). Moreover, some species of Molossidae and Vespertilionidae cohabit with different species in the same roosts.

**Table 3.** Biology features compiled from the literature of bats collected from 2011 to 2015 and bats likely to occur in Campinas, Brazil

Family and species^a^	Forearm (mm)	Weight (g)	Colony size	Feeding habit	Reproduction pattern	Progeny/year	Roosts		Obs.	References
							Artificial	Natural		
Molossidae										
* C. abrasus*	40–51	36–38	7	AI	M	?	LP, RL	PT, TH	–	[5, 28, 50]
* C. planirostris*	29–35	11–12	4–75	GI	P	?	BU, FE, LP, RL	CA, TH	S	[5, 28, 50]
* E. auripendulus*	60–68	23–35	1–15	GI	P	1	RL	RC	–	[5, 28, 53, 54]
* E. bonariensis*	37–49	7–13	10–20	GI	M	1	BR, BU	BT, TH, PT	–	[5, 28, 29, 50, 53]
* E. glaucinus*	56–65	30–47	9–32	AI	P	1	BU, RL	RC, TH	H	[5, 28, 29, 50, 53]
* E. hansae*	37–42	13–17	?	GI	?	?	–	TH, TT	CS	[5, 28, 50, 53]
* E. perotis*	76–83	57	1–100	GI	P	1–2	RL	RC, TH	T, S	[5, 27–29, 50, 53]
* M. temminckii*	27–33	5–7	?	GI	M	1	FE, LP	PT, TH	–	[5, 28, 29, 50]
M. molossus*	38–42	17	1–50	GI	P	1	RL	TH	S	[5, 27, 28, 50]
* M. rufus*	46–53	28–37	1–250	GI	P	1	RL	PT, TH	CS, D	[5, 28, 50]
* N. aurispinosus*	50–52	23	1–3	GI	?	1	AH, RL	CA, RC	–	[5, 28, 50]
N. laticaudatus*	42–47	10	3–3000	AI	M	1	BU, RL	CA, RC	S	[5, 28, 29, 50, 53]
* N. macrotis*	60–65	22–30	9	AI	M	1	BU	RC, FO	–	[5, 28, 29, 50, 53]
* P. nasutus*	48–50	13–14	1–12	GI	?	?	AH, RL	–	S	[5, 27, 28, 50]
T. brasiliensis*	41–45	7–12	<1 × 10^6^	GI	M	1	BU, RL	CA	M	[5, 26–29, 50, 53]
Phyllostomidae										
* A. fimbriatus*	59–71	54	1–30	FR, GI, FL	P	?	AH	FO, FT	TH	[5, 27, 28, 50]
A. lituratus*	>75	>75	4–20	FR, AI	P	1–2	–	FO, TT, PT	D, CS (DR)	[5, 27–29, 49, 50]
* A. obscurus*	55–65	30–39	5–8	FR, PO, NE, FL, GI	P	1	–	FT, TH	–	[5, 27, 28, 50, 53]
* A. planirostris*	48–73	40–69	1–10	FR, PO, NE, GI	P	1	BU	CA, FT, PT, TH	S	[5, 28, 50, 53]
* G. soricina*	32–40	7–17	1–100	NE, PO, FL, GI	P	1–2	AH, BR, BU, CI, MI, TU	CA, RC, TH	S, CS (DR)	[5, 26–29, 49, 50, 53]
* P. discolor*	55–69	26–51	1–25	N, GI, FR, CA	P	?	BU	CA, TH	GF, UG	[5, 26–28, 50]
* P. hastatus*	77–94	64–112	10–100	GI, FR, NE, PO, CA	P	1	BU, RL	CA, FO, PT, TH	S, CS (DR)	[5, 26–29, 49, 50]
* P. lineatus*	43–50	23–26	1–22	FR, GI, NE, PO	P	?	AH, RL	CA, PT	S, CS (DR)	[5, 28, 29, 49, 50]
* P. recifinus*	36–40	14–19	2–20	FR	?	?	–	TH, FO, CA	–	[5, 28]
* S. lilium*	42	21	1–35	FR, PO, NE	P	1	AH, BU	CA, FO, TH	–	[5, 27–29, 50, 53]
* S. tildae*	44–48	21–30	1–10	FR	?	?	–	FO	–	[27, 28]
Vespertilionidae										
E. brasiliensis*	40–46	8–12	1–30	AI	P	?	AH, RL	CA, TH	S (DR)	[5, 28, 49, 50]
E. diminutus*	30–36	5–7	1–10	AI	?	?	BU	TH	CS	[5, 28, 50]
* E. furinalis*	36–42	8	1–20	AI	P	2	BU, RL	TH	CS	[5, 28, 50, 53]
* E. fuscus*	39–57	23	5–700	AI	P	1–2	AH, BU	CA, RC, TH	H, T, M	[26–29, 53]
* H. velatus*	42–50	11	10–65	AI	?	?	RL, BU	–	D	[5, 27, 28, 50, 53]
* L. blossevillii*	36–42	6–10	1	AI	?	3–5	BU	FO, TT	H	[5, 28, 29, 50, 53]
* L. cinereus*	50–57	20–35	1	AI	M	1–4	AH	FO, PT, TH	M	[5, 27–29, 50, 53]
* L. ega*	40–52	10–18	1–20	AI	M	2–4	BU	FO, PT	CS (DR)	[5, 28, 29, 49, 50, 53]
M. albescens*	31–37	7–11	2–20	AI	P	1	AH, BU	CA, RC, TT	–	[5, 28, 50, 53]
* M. levis*	33–41	4–6	1–14	AI	?	?	AH	?	D, CS (DR)	[5, 28, 50]
M. nigricans*	30–36	3–6	1–10	AI	P	<3	AH, BU, RL	CA, PT, RC, TH	CS (DR)	[5, 27, 28, 49, 50, 53]
* M. riparius*	31–38	6–9	?	GI	M	?	BU	–	CS	[5, 28, 53]
* M. simus*	35–41	5–11	?	GI	?	?	RL	BT, TH	CS	[28, 53]

a Underlined species were not identified during the study period but are likely to occur in Campinas.

*RABV positive individuals.

Feeding habit: GI, generalist insectivore; AI, aerial insectivore; FR, frugivore; NE, nectivore; PO, pollinator; CA, carnivore; FL, flower eater.

Reproductive pattern: M, monoestric; P, poliestric.

Artificial roosts: AH, abandoned house; BR, bridge; BU, building; CI, cistern; FE, fence; LP, light pole; MI, mine; RL, roof lining; TU, tunnel.

Natural roosts: BT, banana tree; CA, cave; FO, foliage; FT, forest (rare in urban areas); PT, palm tree; RC, rock crevice; TH, tree hollow; TT, tree top.

Observations: H, hibernation; T, torpor; S, sedentary; M, migratory; D, dispersive (changes roost daily); GF, group foraging; UG, unstable group; CS, cohabits with other species (DR, including *D. rotundus*).

### Temporal distribution of the collected bat specimens

**Fig. 1.**
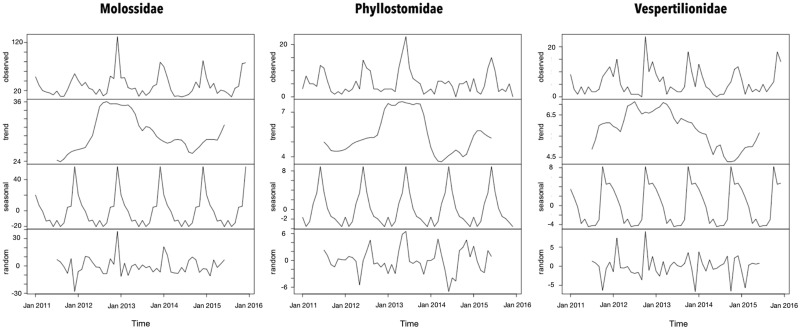
Time-series decomposition of bats collected by a rabies passive surveillance system in Campinas, Brazil.

The seasonal period for the bat records was 1 year according to the periodogram analysis. However, the peaks of Molossidae and Vespertilionidae findings were observed during summer that of Phyllostomidae was observed during winter (Fig. 1). Even though a trend of increased bat records from 2012 and 2013 in all families has been observed, the bat findings were considered stable throughout the time series.

### Spatiotemporal analysis of the collected bat specimens and RABV

**Fig. 2.**
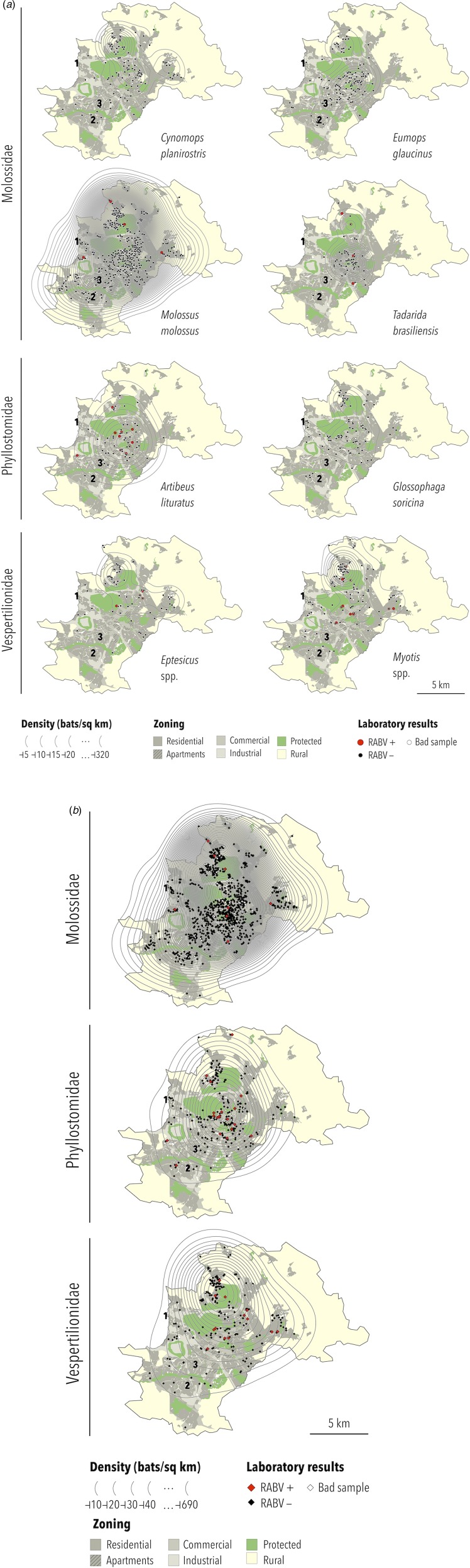
(a) Density, location and RABV diagnosis of selected bat specimens collected by a rabies passive surveillance system from 2011 to 2015 in Campinas, Brazil. Observation: The following domestic animal rabies cases were recorded during the study period: (1) a cat infected with the *Nyctinomops* sp. lineage in 2014; (2) a dog infected with the *D. rotundus* lineage (AgV3) in 2015 and (3) a cat infected with the *Myotis* sp. lineage in 2016. (b) Density, location and RABV diagnosis of selected bat families collected by a rabies passive surveillance system from 2011 to 2015 in Campinas, Brazil. Observation: The following domestic animal rabies cases were recorded during the study period: (1) a cat infected with the *Nyctinomops* sp. lineage in 2014; (2) a dog infected with the *D. rotundus* lineage (AgV3) in 2015 and (3) a cat infected with the *Myotis* sp. lineage in 2016.

Based on the biological features of each collected bat species and those likely to occur in Campinas (Table 3), specimens identified as *Tadarida* sp. were considered *T. brasiliensis*, those as *Glossophaga* sp. were considered *G. soricina* and species from the genera *Epitesicus* (including *Epitesicus* sp.) and *Myotis* (including *Myotis* sp.) were combined. Therefore, six bat species and two genera are represented in thematic maps: *C. planirostris*, *E. glaucinus*, *M. molossus*, *T. brasiliensis*, *A. lituratus*, *G. soricina*, *Eptesicus* spp. and *Myotis* spp. *A. lituratus* was included because this species had the highest RABV prevalence, although the number of collected specimens was below 100. *Nyctinomops* was the only genus with RABV-positive bats (*n* = 1) not represented geographically. The bat specimen density maps are presented in Figure 2a.

The highest density was observed for *M. molossus* (up to 687 specimens per km^2^), and the lowest densities were observed for *G. soricina* and *Eptesicus* spp. (up to 20 specimens per km^2^). In general, the highest density of specimens was obtained at or close to downtown except for *Eptesicus* spp. and *Myotis* spp., which were more frequently observed in the urban–rural interface area of Campinas. For families, the highest density of collected specimens of Molossidae and Phyllostomidae was observed downtown, with Vespertilionidae being observed most frequently in the northwest region of Campinas in the urban–rural interface zone (Fig. 2b).

**Fig. 3.**
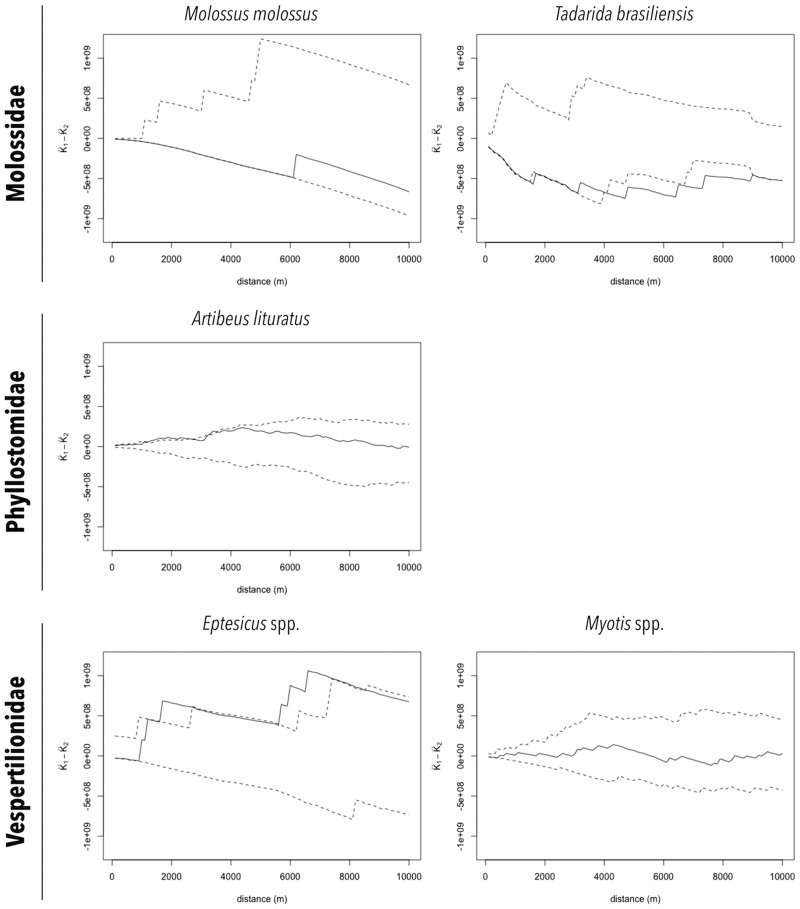
Cluster analysis of RABV diagnosed in bat specimens collected by a rabies passive surveillance system from 2011 to 2015 in Campinas, Brazil. Observation: Solid line represents the empirical K function and dashed lines represent the envelope of the 2.5% and 97.5% quantiles of the K function estimated from 999 Monte Carlo simulations.

The distributions of RABV-positive and RABV-negative samples are also shown in Figures 2a and 2b. In general, no cluster of positive samples was observed within small distances. For *A. lituratus* the empirical K function only barely exceeds the envelope at ~2 km and otherwise remains within the envelope. For *Eptesicus* spp., the empirical K function is only outside the envelope from ~2 to 2.5 km and from 5.75 to 8 km (Fig. 3).

Most of the bats were collected in residential areas (64.2%), in contrast to *T. brasiliensis* (52.9%), which were collected mostly in protected areas (parks and forests) (Table 4) close to downtown.

**Table 4.** Bat specimens collected from 2011 to 2015 and rabies positivity recorded by habitat type in Campinas, Brazil

Bat family/species		Residential single family (188.1 km^2^)	Residential multi-family (7.4 km^2^)	Commercial (40.9 km^2^)	Industrial (46.4 km^2^)	Protected areas (76.3 km^2^)	Public areas (36.3 km^2^)	Rural (400.3 km^2^)	Total (795.7 km^2^)
Molossidae	Samples (%) RABV+ (%)	1058 (57.7) 4 (40)	111 (6) 1 (10)	262 (14.3) 0	33 (1.8) 0	258 (14.1) 4 (40)	105 (5.7) 1 (10)	8 (0.4) 0	1835 (100) 10 (100)
*C. planirostris*	Samples (%) RABV+ (%)	123 (70.3) 0	1 (0.5) 0	18 (10.3) 0	5 (2.9) 0	24 (13.7) 0	4 (2.3) 0	0 0	175 (100) 0
*E*. *glaucinus*	Samples (%) RABV+ (%)	75 (51.4) 0	13 (8.9) 0	37 (25.3) 0	2 (1.4) 0	10 (6.8) 0	9 (6.2) 0	0 0	146 (100) 0
*M*. *molossus*	Samples (%) RABV+ (%)	542 (62.1) 2 (50)	46 (5.3) 0	115 (13.2) 0	9 (1) 0	91 (10.4) 1 (25)	67 (7.7) 1 (25)	3 (0.3) 0	873 (100) 4 (100)
*T. brasiliensis*	Samples (%) RABV+ (%)	12 (8.6) 2 (40)	0 0	30 (21.4) 0	1 (0.7) 0	74 (52.9) 2 (40)	1 (0.7) 0	0 0	140 (100) 5 (100)
Phyllostomidae	Samples (%) RABV+ (%)	156 (48) 9 (42.9)	0 0	50 (15.4) 3 (14.3)	7 (2.1) 0	60 (18.5) 5 (23.8)	24 (7.4) 0	3 (0.9) 2 (9.5)	325 (100) 21 (100)
*A. lituratus*	Samples (%) RABV+ (%)	29 (40.3) 4 (33.3)	6 (8.3) 1 (8.4)	19 (26.4) 3 (25)	1 (1.4) 0	11 (15.3) 4 (33.3)	6 (8.3) 0	0 0	72 (100) 12 (100)
*G. soricina*	Samples (%) RABV+ (%)	67 (61.5) 0	6 (5.5) 0	10 (9.2) 0	4 (3.7) 0	10 (9.2) 0	11 (10.1) 0	1 (0.8) 0	109 (100%) 0 (0%)
Vespertilionidae	Samples (%) RABV+ (%)	256 (73.6) 6 (42.9)	5 (1.4) 1 (7.1)	24 (6.9) 2 (14.3)	2 (0.6) 0	25 (7.2) 4 (28.6)	26 (7.5) 1 (7.1)	10 (2.8) 0	348 (100) 14 (100)
*Eptesicus* spp.	Samples (%) RABV+ (%)	68 (67.3) 2 (50)	1 (1) 0	11 (10.9) 1 (25)	0 0	6 (5.9) 1 (25)	10 (9.9) 0	5 (5) 0	101 (100) 4 (100)
*Myotis* spp.	Samples (%) RABV+ (%)	136 (78.2) 4 (40)	3 (1.7) 1 (10)	4 (2.3) 1 (10)	0 0	15 (8.6) 3 (30)	12 (6.9) 1 (10)	4 (2.3) 0	174 (100) 10 (100)
Total	Samples (%) RABV+ (%)	1470 (58.6) 19 (42.2)	141 (5.6) 4 (8.9)	336 (13.4) 5 (11.1)	42 (1.7) 0	343 (13.7) 13 (28.9)	155 (6.2) 2 (4.4)	21 (0.8) 2 (4.4)	2508 (100) 45 (100)

RABV+, rabies positive samples.

Obs.: 29 bats had no family, genus and species identified.

**Fig. 4.**
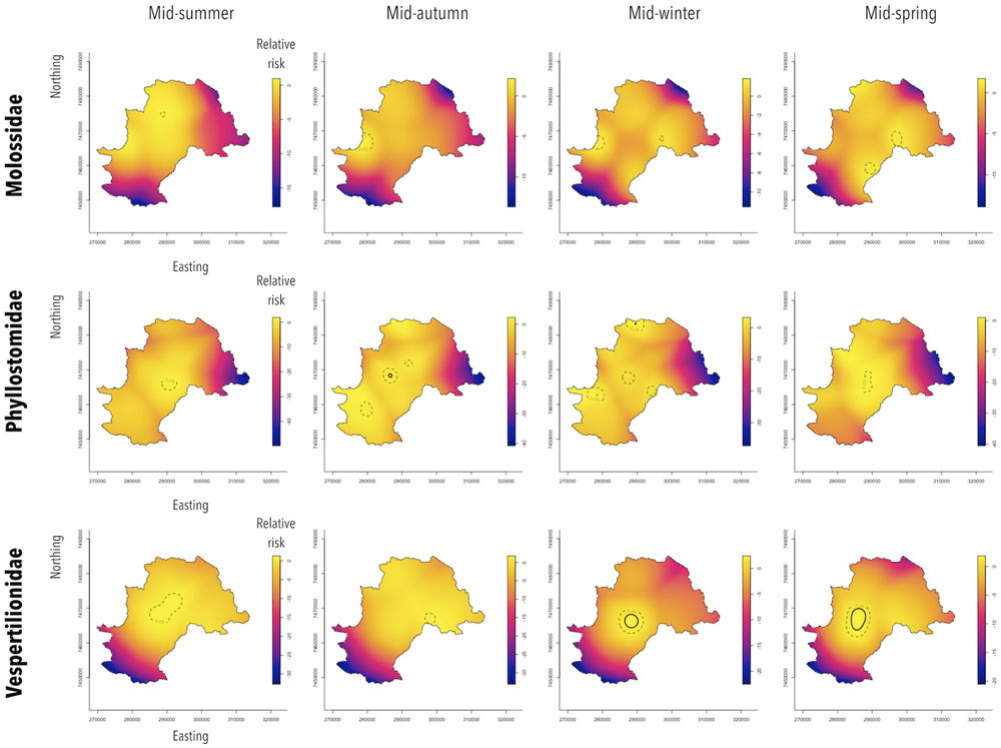
Spatiotemporal relative risk of RABV-positive bats from specimens collected by a rabies passive surveillance system in Campinas, Brazil. Observation: Solid circles represent areas with excess of relative risk of rabies positivity with 0.1% of significance and dashed circles, with 5% of significance.

The bat families behaved differently in terms of their seasonal and spatial patterns (Fig. 4). Spatiotemporal analysis showed that in Molossidae, a significant excess of relative risk of rabies positivity (*P* < 0.05) was observed in peripheral areas, in the western region from autumn to winter and in the eastern region of Campinas from winter to spring. During summer, a small area of relative risk excess was observed in the northern region of the city. For Phyllostomidae, a significant excess of relative risk (*P* < 0.05) was observed downtown throughout the year, which intensified during autumn (*P* < 0.001). Moreover, from autumn to winter, an excess of relative risk (*P* < 0.05) was also observed in peripheral zones of northern and southern areas of Campinas. Finally, Vespertilionidae showed a more stable area of significant relative risk excess (*P* < 0.05) throughout the year at downtown, which intensified from winter to spring (*P* < 0.001).

## Discussion

The current work was based on records of a passive bat rabies surveillance system in the city of Campinas, Brazil. The findings showed that bat family densities varied in space and time. Moreover, due to the location and situation at which bats were collected, the risk of RABV transmission to domestic animals and humans was high, as recent reports of two cats and a dog infected with bat-associated RABV shown. The bat rabies surveillance is crucial to prevent the transmission of bat-associated RABV to domestic animals in urban areas, especially after the recent declaration of AgV1- and 2-free area by the Brazilian government. This situation will in turn halt the mass vaccination of dogs and cats and promote susceptibility to bat-associated RABV.

Only dead or possibly ill insectivore bats were collected in this study and no active surveillance was conducted. Therefore, proper inferences about the bat population distributions and dynamics were not possible. Moreover, all discussion is focused on bats collected in the urban area of Campinas [16]. Few bats from rural areas were collected since the rabies surveillance system performed by animal health authorities is specific but passive and only 2% of the Campinas population lives in these areas [16]. All areas of the city, particularly the peripheral zones, are systematically informed by UVZ about RABV infection from bats. Therefore, differences in specimen densities were not caused by the performance heterogeneity of the rabies surveillance system. The proportions of collected bat species were similar to those in other studies in Campinas [15] and elsewhere in Brazil [5]. No haematophagous bats were collected in this study, although one dog was infected with RABV AgV3 (*D. rotundus* lineage) in 2015. In recent years, RABV lineages similar to AgV3 have been identified in *A. lituratus* [13, 33, 34].

Regarding the location of the recorded specimens, most were found inside households. Molossidae was the primary contributor, with the highest proportions in the genera *Tadarida* and *Nyctinomops*, followed by *Molossus*, with the latter being the most frequently collected genus. Most of the RABV-positive samples from this genus were obtained in residential areas. Although *Cynomops* and *Eumops* showed similar proportions, no RABV-positive samples were detected. *Tadarida* was an exception because these bats were collected mostly in parks and protected areas downtown.

In Molossidae, most of the specimens were captured alive, particularly *Tadarida* and *Nyctinomops*, which is a concerning fact, particularly for cats. Domestic cats are extremely attracted to moving objects and animals [35, 36], and are therefore exposed to increased risk for rabies infection by Molossidae inside households. In Phyllostomidae and Vespertilionidae, most specimens were captured dead outside the households, and unsupervised or feral cats could have been exposed to or have actually preyed upon those bats.

Vespertilionidae were more frequently captured in residential areas, where most of its RABV-positive samples were found. Most Phyllostomidae were also found in residential areas but in a lower proportion and were also collected in commercial and protected and public areas, where most of the RABV-positive samples were found. *A. lituratus* was the species with the highest RABV occurrence. Phyllostomidae were more densely found downtown, but Vespertilionidae were observed in urban–rural zones in peripheral areas of Campinas.

Although the seasonality period of the three families was the same (1 year), peaks of Molossidae and Vespertilionidae findings were observed during summer, whereas the peak of Phyllostomidae was during winter. Molossidae were more frequently collected downtown, but the excess of relative risk for RABV infection was observed in peripheral zones of Campinas, which could be explained by analysing each species individually. *M. molossus*, the most abundant species in the sample, is sedentary, forming small colonies, but it might be the species that most benefited in the urban area of Campinas among all the collected species. The species is very adapted to man-made structures, particularly roof linings. Peak findings of these bats (mostly alive) were observed during summer, indicating that although the bats benefited from the increased density of insects attracted by city lights. Even so, they might have been marginally affected by pollution [37], pesticides [38] or even (sometimes illegal) control by the inhabitants. Moreover, city lights can directly affect bat biology [20, 39].

Although composing only approximately the equivalent of 10% of the *M. molossus* sample and with no RABV-positive individuals being observed in the sample, *Molossus rufus* are notable because they are dispersive bats and may share roosts with other species, including *M*. *molossus* [40]. Interspecific contact, particularly the contact between haematophagous and non-haematophagous bats, is an important epidemiological parameter that is not properly studied. However, because *D*. *rotundus* can also use man-made structures, such as roof linings, and eventually roosts with other species [41–45], these bats may be responsible for infecting *Molossus* in rural areas. Moreover, Vespertilionidae species, such as *Eptesicus* sp. and *Myotis* sp., in which RABV-positive samples were observed, can also roost with other species, including *D*. *rotundus*, and showed high densities of findings in peripheral areas of Campinas. Although less susceptible to RABV infection from other species, *M*. *molossus* may be an important reservoir in peripheral areas and less important in urban areas despite the high density of findings downtown.

*T. brasiliensis* showed a more restricted distribution of findings than *M*. *molossus*, but was also observed downtown. This species is migratory and can form very large colonies, the largest among the species collected in this study (up to 1 000 000 individuals) [46]. These bats can also roost in man-made structures, including roof linings, but were more frequently collected in parks and protected areas downtown. Unlike *M*. *molossus*, a high prevalence of RABV was observed, being the most important reservoir among Molossidae. The mass mortality of *Tadarida* due to RABV infection has been reported elsewhere [47, 48], and the health authorities of Campinas should thus continuously investigate this species. Among the Molossidae collected in this study, *T*. *brasiliensis* should be considered the species with the highest capacity for introducing and maintaining different RABV lineages because of its capacity for migrating and forming colonies with numerous individuals. Compared with *M. molossus*, this bat might be more resilient to the urban environment because fewer specimens were collected by the passive surveillance system despite its high abundance.

Although few Nyctinomops were collected, one sample tested RABV-positive and one reintroduction event in cats occurred in 2014 by a virus lineage specific to this species in Campinas. This species should also be monitored by the rabies surveillance system.

Phyllostomidae were more frequently collected dead during winter. Although Phyllostomidae exhibit generalist feeding habits, insects are the primary item in the diet of this family, followed by fruits, nectar, flowers, pollen and other invertebrate animals [26–29, 49–51]. Those food sources are all scarce during the winter, which is more likely the primary cause of the highest mortality in that season. Of the bat families collected in this study, Phyllostomidae was the least capable of using man-made structures and was also is the family that had the lowest proportion of specimens collected in residential areas. Thus, this family benefited least in the urban area of Campinas. Moreover, RABV prevalence was higher in Phyllostomidae (6.5%) than in the other families, particularly in *A. lituratus*, with the highest prevalence (16.7%) among all collected species and the only species in its family with RABV-positive samples. This result might have been caused by two factors, listed as follows: (a) Phyllostomidae exhibit reduced fitness because they are larger and heavier, requiring a more critical energy balance. Moreover, all collected species of this family are poliestric, and females are therefore more susceptible to seasonal changes in climate and food availability; (b) dispersal behaviour, with a daily change in the roost by females [52], may increase the contact with other individuals, including other species. *A. lituratus* roosts preferably in treetops and palm trees, and from the collected species, few used the same roosts, including other *Artibeus* and *Myotis albescens*, which had the second largest RABV prevalence in the sample. However, because of the high RABV prevalence and dispersal behaviour, *A. lituratus* should also be a target species in the rabies surveillance system.

Like Molossidae, Vespertilionidae also showed peaks of findings during summer. This family has different biological features; for example, most species roost with other species, including *D*. *rotundus*. Nearly all species feed on aerial insects, which are the most abundant in summer. Vespertilionidae were the smallest of the specimens collected in Campinas, but most of the species are poliestric and produce large progenies (in general, over two pups), making the energy balance critical and increasing the susceptibility to seasonal climatic changes. Vespertilionidae are the family most capable of using man-made structures and apparently the family that most benefit in the urban environment of Campinas. Although more frequently collected in peripheral areas, excess relative risk for RABV infection was observed downtown throughout the year, which intensified from winter to spring. This observation could be explained by the increased fitness in the urban environment and increased competition for food and roosts during the winter. For those reasons, *Eptesicus* sp. (particularly *E*. *brasiliensis*, which is sedentary) and *Myotis* sp., should be target species in the rabies surveillance system because they are more likely to have contact with other insectivore and haematophagous bat species and are therefore possibly responsible for the maintenance and dissemination of different RABV lineages. One of the RABV reintroduction events in cats was due to a *Myotis* lineage in 2016.

Finally, RABV was not diagnosed in the two abundant species of Molossidae, *C. planirostris* and *E. glaucinus*. Both species showed a high density of findings downtown. Both form small colonies, but *C*. *planirostris* is smaller and lighter, roosting on fences and light poles, and is sedentary. *E. glaucinus* is more adaptable to man-made structures and is one of the few species collected in Campinas that hibernates. No specific biological features explained why RABV was not diagnosed in these species.

Although only dogs were recorded to contact RABV-positive bats in 2015, cats should not be neglected in educational or strategic vaccinations. The probability of infectious contact between bats and domestic animals was previously calculated in Campinas, showing that such contacts are at least twofold higher in cats [15]. All the information available regarding bat fauna and RABV in urban areas is essential for the new challenges presenting with the declaration of RABV AgV1- and AgV2-free zones in Brazil by the Ministry of Health in 2015. This measure will end the mass vaccination of dogs and cats against rabies, most likely making most susceptible to bat or wildlife RABV. Moreover, education, awareness and when required, strategic dog and cat vaccinations should be prioritised to properly address this situation.
